# Population genetic structure of gray wolves (*Canis lupus*) in a marine archipelago suggests island-mainland differentiation consistent with dietary niche

**DOI:** 10.1186/1472-6785-14-11

**Published:** 2014-06-10

**Authors:** Astrid V Stronen, Erin L Navid, Michael S Quinn, Paul C Paquet, Heather M Bryan, Christopher T Darimont

**Affiliations:** 1 Mammal Research Institute, Polish Academy of Sciences, ul. Waszkiewicza 1, Białowieża 17-230, Poland; 2 Department of Biotechnology, Chemistry and Environmental Engineering, Aalborg University, Sohngaardsholmsvej 57, Aalborg 9000, Denmark; 3 Faculty of Environmental Design, University of Calgary, 2500 University Dr. NW, Calgary, Alberta T2N 1 N4, Canada; 4 Institute for Environmental Sustainability, Mount Royal University, 4825 Mount Royal Gate SW, Calgary, Alberta T3E 6 K6, Canada; 5 Department of Geography, University of Victoria, PO Box 3060 STN CSC, Victoria, British Columbia V8W 3R4, Canada; 6 Raincoast Conservation Foundation, PO Box 86 Denny Island, British Columbia V0T 1B0, Canada; 7 Hakai Beach Institute, Box 309, Heriot Bay, British Columbia V0P 1H0, Canada

**Keywords:** *Canis lupus*, Ecological divergence, Marine resources, Niche, Population genetic structure, Traditional ecological knowledge, Wolf

## Abstract

**Background:**

Emerging evidence suggests that ecological heterogeneity across space can influence the genetic structure of populations, including that of long-distance dispersers such as large carnivores. On the central coast of British Columbia, Canada, wolf (*Canis lupus* L., 1758) dietary niche and parasite prevalence data indicate strong ecological divergence between marine-oriented wolves inhabiting islands and individuals on the coastal mainland that interact primarily with terrestrial prey. Local holders of traditional ecological knowledge, who distinguish between mainland and island wolf forms, also informed our hypothesis that genetic differentiation might occur between wolves from these adjacent environments.

**Results:**

We used microsatellite genetic markers to examine data obtained from wolf faecal samples. Our results from 116 individuals suggest the presence of a genetic cline between mainland and island wolves. This pattern occurs despite field observations that individuals easily traverse the 30 km wide study area and swim up to 13 km among landmasses in the region.

**Conclusions:**

Natal habitat-biased dispersal (i.e., the preference for dispersal into familiar ecological environments) might contribute to genetic differentiation. Accordingly, this working hypothesis presents an exciting avenue for future research where marine resources or other components of ecological heterogeneity are present.

## Background

Recent evidence indicates that ecological and environmental variation can result in genetic differentiation within many taxa, including highly mobile species. Examples include sea turtles (reviewed in Bowen and Karl [1]), fish species such as herring (*Clupea harengus* L., 1758; André et al. [2]) and hake (*Merluccius merluccius* L., 1758; Milano et al. [3]), and mammal species including the orca (*Orcinus orca* L., 1758; Hoelzel et al. [4]), cougar (*Puma concolor* L., 1771; McRae et al., [5]), lynx (*Lynx canadensis* Kerr, 1792; Rueness et al., [6]), coyote (*Canis latrans* Say, 1823; Sacks et al. [7]), and wolves (*C. lupus* L., 1758; Musiani et al. [8]; Pilot et al. [9]; Weckworth et al. [10]–[12]). For example, Muñoz-Fuentes et al. [13] showed strong genetic divergence over distances less than 500 km between wolves of coastal and interior regions of British Columbia (BC), Canada. Ecological and environmental dimensions such as climate and prey availability between areas, not distance, best explained population structure. These patterns arise because individuals may be more likely to survive and reproduce within their natal habitats (Davis and Stamps [14], Nosil et al. [15], Edelaar et al. [16]), which, in turn, can influence population genetic structure. A prediction from this body of work is that genetic divergence might be detected even over short geographical distances, and for highly mobile animals, should there be a sharp gradient in environmental conditions.

Such sharp ecological transitions occur between mainland and adjacent island environments within coastal BC. Although distances between mainland and neighbouring islands are small (<1500 m), the environments have striking geological and ecological differences. The mainland is topographically rugged, contains less shoreline for a given area and is relatively species-rich. In contrast, the neighbouring islands are less mountainous, have more complex shorelines, and host fewer species; notably absent are grizzly bears, (*Ursus acrtos horribilis* Ord, 1815), which compete with wolves for marine resources (Darimont and Paquet [17]; Paquet et al. [18]). Owing to these different environments, analyses of faeces and stable isotope data have identified distinctly different realized niches. Wolves from island populations rely on marine resources for up to 85% of their diet, whereas mainland conspecifics rarely include more than 30% (Darimont et al. [19,20]). Additionally, the coastal mainland supports moose (*Alces alces* L., 1758) and mountain goats (*Oreamnus americanus* Blainville, 1816) that are absent or rare on coastal islands. Consequently, these major prey items are commonly detected in wolf diet in mainland areas and only very rarely on islands (Darimont et al. [21]). Moreover, likely reflecting these distinct habitat and dietary niches, parasite prevalence also differs between areas; there is higher faecal prevalence of *Giardia* sp. infections on islands and a lower prevalence of *Diphyllobothrium* sp. relative to mainland sites (Bryan et al. [22]).

Our objective was to examine genetic data from wolves of coastal BC over a limited geographic area (~2000 km^2^, with a generally east–west mainland-island axis of <30 km) to test the hypothesis that ecological heterogeneity can drive population genetic structure of a highly mobile animal within a small area. We note that this prediction was also informed by holders of traditional ecological knowledge (TEK) in the Heiltsuk First Nation area, who distinguish between mainland “timber wolf” and island “coastal wolf” forms. Given these scholarly- and TEK-informed hypotheses and the sharp environmental gradients on the BC coast, we expected mainland-island genetic differentiation that mirrors ecological differences among neighbouring social groups.

## Methods

### Study area

The central coast of BC is a remote network of islands and naturally fragmented mainland landmasses with limited (but increasing) industrial anthropogenic disturbance. The area is characterized by a wet and temperate climate, and annual precipitation typically exceeds 350 cm (Darimont and Paquet [17]). A core area (~2000 km^2^) centered on Bella Bella (52°10’ N, 128° 09’ W) served as the location for this study (Additional file 1). This landscape is surrounded by ocean, which separates a mainland landmass (823 km^2^) and five main islands ranging in size from 150–250 km^2^. Distances from island to mainland range from 250 m to 1450 m. Observational and genetic data (Darimont et al. [19]; Navid [23]) suggest that wolf packs, defined by the multi-year association of genetically and morphologically distinct individuals, have either island or mainland home ranges. However, one group (Yeo-Coldwell [YC]) primarily uses island habitat but also a portion of the adjacent mainland. Other units are either mainland groups (Upper Roscoe [UR], Lower Roscoe [LR] or island groups (Cunningham-Chatfield [CC], Denny-Campbell [DC]). Moreover, wolves are commonly observed swimming among landmasses, and home ranges of social groups often include multiple islands or mainland landmasses (e.g. peninsulas; Paquet et al. [18]; Darimont [24]; McAllister and Darimont [25]).

### Sampling

One thousand and seventy-four (1074) wolf faecal samples were collected between winter 2003 and winter 2004. We collected the following number of samples per season: spring: n = 416 summer: n = 297 fall: n = 292 winter: n = 69. Sampling areas included wildlife trails, logging roads, and electrical power rights-of-way. We preserved each sample in a 50-ml Falcon tube with 95% ethanol. We selected samples for genetic analysis based on characteristics of the samples and collection sites that best predicted amplification success (minimal physical decay, high moisture content, canopy cover; Navid [23]). We extracted DNA from faecal samples with Qiagen QIAamp® DNA Stool Mini Kits and the ‘Protocol for isolation of DNA from larger amounts of stool’ (QIAamp® DNA Stool Mini Kit handbook, http://www.qiagen.com/literature/). We performed DNA extractions in a room physically separated from amplified PCR products and used exclusively for this study to reduce the risk of contamination. Final purified extracts were refrigerated at +4°C until use.

### Microsatellite amplification

We amplified a panel of 14 microsatellite markers (13 autosomal and one Y chromosome marker). These were *FH2001, FH2010, FH2017, FH2054, FH2088, FH2096, FH2422* (Breen et al. [26]), *FH3313, FH3725* (Guyon et al. [27]), *PEZ06, PEZ08, PEZ15, PEZ19* (Halverson J. in Neff et al. [28]), and the Y-chromosome marker *MS41B* (Sundquist et al. [29]). We genotyped n = 477 faecal samples. Polymerase chain reaction (PCR) conditions optimized for the markers, based on the Qiagen multiplexing kit, were: initial denaturation at 95°C for 15 minutes, then 35 cycles of denaturation at 94°C for 30 sec, annealing at 58°C for 90 sec, extension at 72°C for 60 sec, with final extension at 60°C for 30 min. Organisation of markers into multiplexes is shown in Additional file 2. Amplified PCR product was loaded into a 6.5% denaturing polyacrylamide gel, and run on a LICOR4300s DNA analyzer. Genotyping was done with a LICOR’s SAGA GT version 3.3 microsatellite analysis software.

We accepted for further analyses samples that amplified at least 9/14 loci, and used the Excel Microsatellite Toolkit (Park [30]) to test for the presence of matching profiles. We consolidated matches (i.e., profiles with ≥ 75% matching alleles to account for uncertainty in genotyping) into one profile and retained the profile with the highest amplification rate. Matches were tested across all samples, but only observed within wolf groups and 33 profiles were removed.

N = 116 individual profiles were identified. Only 28 wolves (24%) were identified as males, and we observed six alleles for marker *MS41B* (*209, 211, 213, 217, 219, 221*). We used MICRO-CHECKER 2.2.3 (van Oosterhout et al. [31]) to assess possibilities of null alleles, large allele dropout, and scoring errors due to stutter peaks. We repeated genotyping of 50 samples collected during the fall season (deemed to represent 50 different individuals from all four groups [CC, DC, LR, YC] based on the abovementioned criteria) to evaluate data quality and estimate genotyping error. Here we estimated per-locus error rates based on the percentage of loci that did not show the same result twice (Additional file 3). Loci for which we obtained the same results twice were accepted as duplicated loci. From these results we obtained duplicate genotypes comprising five or more loci for 18 individuals (i.e. every locus in each of these 18 genotypes provided consistent results when re-tested). Based on amplification and error rates (Additional file 3) we removed *MS41B, PEZ08, FH2017*, and *FH3313* from further analyses.

### Statistical analyses

We calculated allelic diversity and observed and expected heterozygosity (with correction for sample size bias; Nei [32]) per locus in GENEPOP 3.4 (Raymond and Rousset [33]) and Genetix 4.05.2 (Belkhir et al. [34]), and F_IS_ according to Weir and Cockerham [35], for mainland and island wolves. We tested for departures from Hardy-Weinberg equilibrium per locus in GENEPOP 3.4 with the Markov chain method (Guo and Thompson [36]). The results were adjusted to account for multiple comparisons with the false discovery rate (FDR, Verhoeven et al. [37]). Subsequently, we performed centered and scaled principal component analyses (PCA) with the *adegenet-*package (Jombart [38]) in R 2.14.2 (R development Core Team [39]). The PCA approach does not assume genetic equilibrium conditions and is well-suited for identifying spatial patterns such as genetic clines (i.e., gradients rather than separate clusters or complete admixture) that can be difficult to detect (Jombart et al. [40]). We repeated the PCA with the 18 individuals for which we had duplicated genotypes (and thus higher confidence), and all wolves identified as males to confirm the presence of the observed cline. On average, male wolves may disperse longer distances than females and are more likely to join new packs. As our data set comprised relatively few confirmed males, we tested these results separately to check if the island-mainland gradient remained consistent. A high proportion of females in our sample might otherwise have contributed to the observed gradient if females disperse less frequently and/or shorter distances than males. We then performed a spatial PCA (henceforth sPCA; Jombart et al. [40]), which also takes spatial sampling information into account. As multiple samples were at times collected from the same location, we added 100 m of jitter (small amount of noise) to the UTM coordinates. We performed a spatial autocorrelation in GenAlEx (Peakall and Smouse [41] and references therein) to examine the possible existence of isolation-by-distance in our data set. We used distance classes of 5 km to obtain fine-scale results for our study area. Finally, we performed a partial Mantel-test in R with the *Vegan* package (Oksanen et al. [42]) to examine the relationship between genetic distance and island-mainland habitat type while controlling for geographic distance. This allowed us to test whether there was an effect of habitat type on fine-scale genetic structure after accounting for the effect of geographic distance. Geographic distance and habitat may be co-linear and their effects could be difficult to separate. Consequently, we also examined the relationship between geographic distance and habitat type. For these tests we incorporated co-dominant genotypic and Euclidean geographic distance matrices exported from GenAlEx and a third matrix with island-mainland habitat designations. We used Pearson’s correlation coefficient with n = 999 permutations.

## Results

The average number of alleles per locus was 5.8 for mainland wolves and 6.8 for island wolves (Table 1). For mainland wolves, expected heterozygosity was 0.632 and five loci showed departures from Hardy-Weinberg equilibrium with observed levels of heterozygosity lower than expected. F_IS_ results were positive for all loci with a mean value of 0.264. For island wolves, expected heterozygosity was 0.690 and seven loci showed departures from Hardy-Weinberg equilibrium (four of these were consistent between mainland and island wolves). F_IS_ results for island wolves were positive for all except two loci, with a mean value of 0.211. We identified possible null alleles and stutter peaks for the overall sample, but dropout of large alleles was not detected (Additional file 3).

**Table 1:** **Genetic diversity measures for wolves ( *Canis lupus *) from the central coast of British Columbia, Canada**

**Locus**	**#AllelesMA/IS**	**H**_**o **_**MA**	**H**_**e **_**MA**	***P*-value + S.E. MA**	**F**_**IS **_**MA**	**H**_**o **_**IS**	**H**_**e **_**IS**	***P*-value + S.E. IS**	**F**_**IS **_**IS**
FH2054	6/8	0.465	0.595	0.016 + (0.0035)	0.220	0.456	0.720	**0.000 + (0.0000)**	0.368
FH2001	6/10	0.432	0.715	**0.000 + (0.0000)**	0.399	0.789	0.811	0.022 + (0.0069)	0.028
FH2096	4/3	0.318	0.334	0.171 + (0.0081)	0.047	0.592	0.502	0.276 + (0.0054)	-0.180
FH2010	3/6	0.211	0.319	0.023 + (0.0018)	0.342	0.355	0.538	**0.000 + (0.0004**)	0.342
FH2088	5/5	0.614	0.694	0.106 + (0.0050)	0.116	0.761	0.726	0.071 + (0.0054)	-0.049
FH2422	8/7	0.561	0.828	**0.000 + (0.0000)**	0.325	0.574	0.734	**0.000 + (0.0001)**	0.220
PEZ06	9/9	0.421	0.811	**0.000 + (0.0000)**	0.484	0.409	0.831	**0.000 + (0.0000)**	0.510
PEZ19	3/3	0.371	0.550	0.028 + (0.0021)	0.328	0.362	0.585	**0.003 + (0.0005)**	0.384
PEZ 15	8/12	0.605	0.796	**0.000 + (0.0000)**	0.243	0.452	0.755	**0.000 + (0.0000)**	0.403
FH3725	6/5	0.636	0.683	**0.000 + (0.0001)**	0.068	0.647	0.702	**0.000 + (0.0002)**	0.079
MEAN	5.8/6.8	0.463	0.632		0.264	0.539	0.690		0.211

Genetic variation across 10 microsatellite loci for individuals from mainland (MA, n = 44) and island (IS, n = 72) areas.

H_e_ values are calculated with correction for uneven samples sizes (Nei [32]).

*Bold font indicates significant *p*-values.

**Figure 1:**
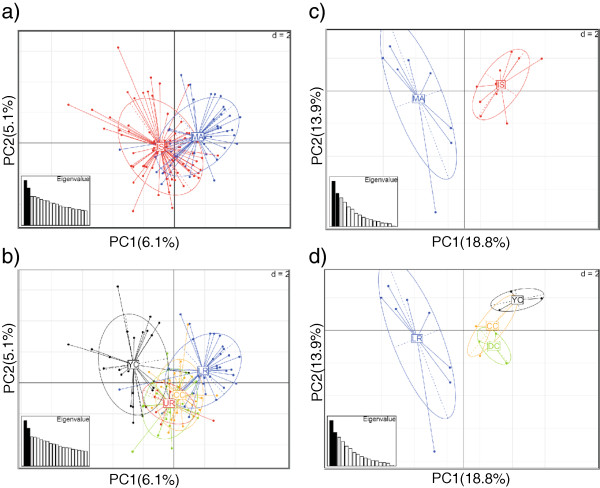
**Principal component analyses of wolves (*Canis lupus*) from the central coast of British Columbia, Canada showing geographic distribution of individuals. a)** Individual (*n* = 116) profiles based on ≥ 10 microsatellite loci labelled according to mainland (MA) and island (IS) sample locations. **b)** Individual profiles (*n* = 116) based on ≥ 10 microsatellite loci labeled according to membership in five wolf family groups: Upper Roscoe (UR) and Lower Roscoe (LR) on the mainland, and Yeo-Coldwell (YC), Cunningham-Chatfield (CC), and Denny-Campbell (DC) islands. Note that the label for DC (green colour) is overlapped by UR (red colour). **c)** A subsample of individual profiles (*n* = 18) with duplicated genotypes based on ≥ 5 loci labelled according to mainland and island sample locations. **d)** Individual profiles (*n* = 18) with duplicated genotypes based on ≥ 5 loci labelled according to membership in four wolf family groups LR, YC, CC, and DC (none from UR).

**Figure 2:**
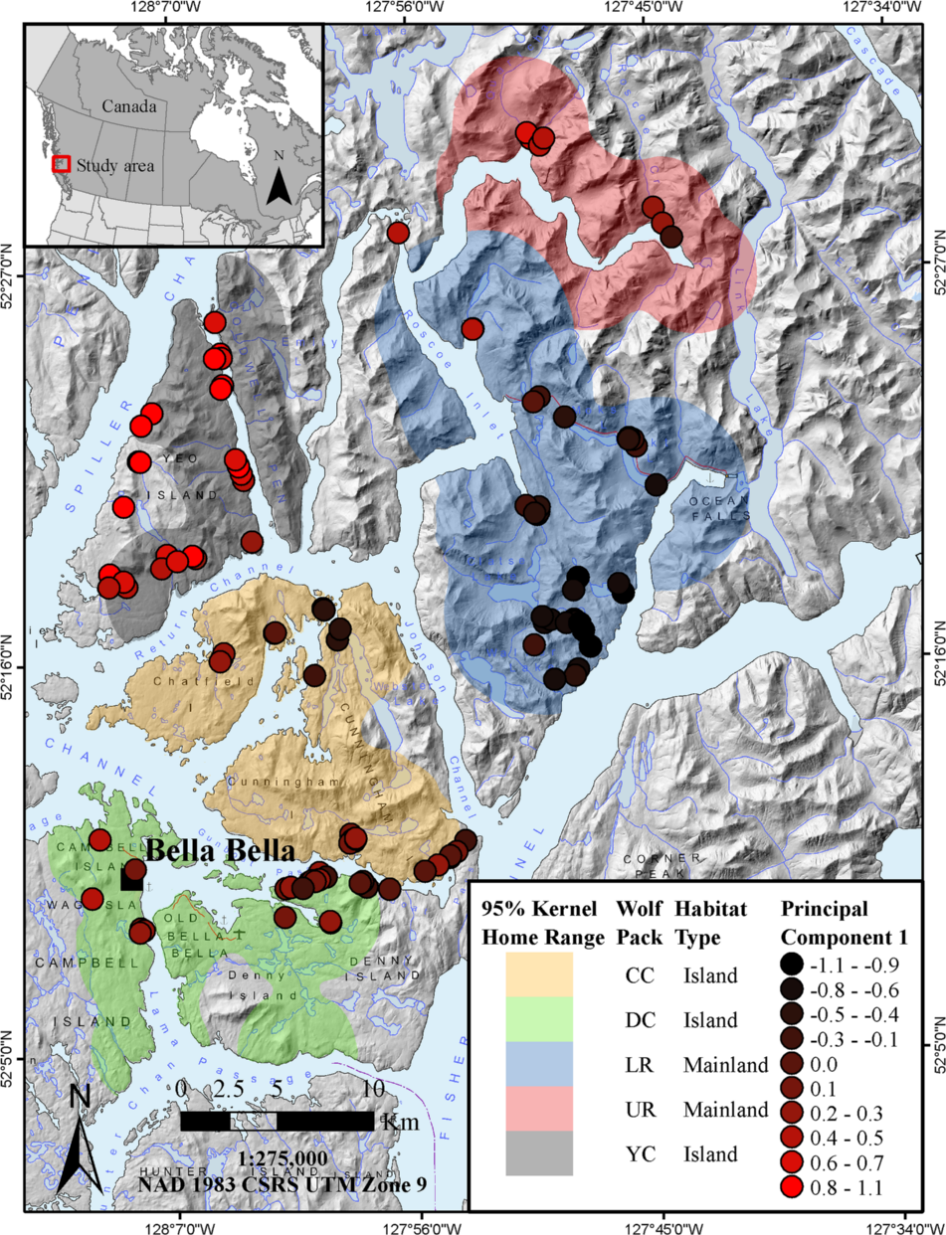
**Spatial principal component analysis of wolves (*Canis lupus*) from the central coast of British Columbia, Canada, showing the first global structure mapped across the study area.** Individual profiles (*n* = 116) are based on ≥ 10 microsatellite loci and originate from five wolf family groups: Upper Roscoe (UR) and Lower Roscoe (LR) on the mainland, and Yeo-Coldwell (YC), Cunningham-Chatfield (CC), and Denny-Campbell (DC) islands.

PCA results indicated the presence of a genetic cline between island and mainland wolves (Figure 1a, c). Although overlap was extensive, the results suggested an east–west gradient in profiles across < 30 km. Examination of genetic profiles based on the known wolf groups in the area (UR was not represented in the second analysis) suggested limited overlap between LR (Mainland) and YC (Island) wolves (Figure 1b, d). The CC and DC island groups occupy an intermediate position, along with the UR group from the mainland. Colour plots (Additional file 4) show the individual genetic profiles throughout the study area, and display a similar east–west gradient from the mainland to the islands. The PCA results for individuals identified as males (n = 28) were consistent with island-mainland differentiation (Additional file 5). For the sPCA, one global structure (and no local structure) was apparent (Additional file 6). When mapped across the geographic space, the global structure revealed an east–west gradient where YC and LR were the most differentiated groups (Figure 2). The partial Mantel test gave a correlation coefficient of 0.011 (p-value 0.351) between genetic distance and habitat matrices. The test between geographic distance and habitat matrices produced a correlation coefficient of 0.568 (p-value 0.001). Spatial autocorrelation results were positive for the first 17 km, negative from approximately 17–45 km, and subsequently positive (though this may be considered as zero autocorrelation at the larger distance classes with wide confidence intervals; Additional file 7).

## Discussion

### Genetic variation

Allelic diversity and expected heterozygosity for island wolves (6.8, 0.690) and mainland wolves (5.8, 0.63) were relatively high and comparable to values reported for island populations of wolves on the Pacific Coast in southeast Alaska (5, 0.52; Weckworth et al. [10]) and coastal island populations in Arctic Canada (4.2, 0.61; Carmichael et al. [43]). Allelic diversity and expected heterozygosity were somewhat lower for wolves on the mainland portion of our study area, although this might, at least in part, reflect chance effects of our relatively small sample sizes. Comparison with F_IS_ values from southeast Alaska islands (0.05) and coastal islands in Arctic Canada (0.181) suggest a higher degree of mating among relatives in mainland (0.264) and island (0.211) wolves from coastal BC. However, Carmichael et al. [43] also observed high F_IS_ values on Victoria Island (0.427, n = 52) and on islands in the High Arctic (0.629, n = 11). Based on the findings from Alaska wolves, continental wolves appear to have higher genetic diversity. We would also expect a similar situation for our study area, as mainland wolves have a wider surrounding area from which to receive immigrants. However, there are known wolf groups on neighbouring islands not included in this study and we cannot exclude the possibility that immigration from these areas may have augmented the diversity in our sample of island wolves.

### Non-invasive sampling and genotyping

Allelic dropout in non-invasive sampling (Santini et al. [44]) could, at least in part, explain the lower values for observed heterozygosity, the high number of loci not in Hardy-Weinberg equilibrium, and the positive F_IS_ values. Our results could also have been influenced by the presence of null alleles. When most of the loci indicate null alleles, however, the MICRO-CHECKER program warns there may not be random mating in the population (panmixia). The PCA and sPCA findings of island-mainland differentiation suggest absence of panmixia in our study area. We therefore believe that island-mainland structure contributed to the frequent reports of null alleles. The study area is difficult to access, and many samples may have been several weeks old and thus affected by exposure to the humid climate (Santini et al. [44]; Navid [23]). Our results are based on analyses of faecal material, where duplicated genotypes were obtained for 15% (18 of 116) individuals. Error rates were high, but we do not expect any consistent bias between areas. Results from the duplicated genotypes accord with the larger dataset, although further sampling and multiple-tube analyses (e.g. Santini et al. [44]) would be necessary for accurate identification of individual wolves and to confirm dispersal events in our study area.

### Evolutionary ecology and genetic differentiation between mainland and island wolves

The partial Mantel test showed no significant relationship between genetic distance and island-mainland habitat type when accounting for geographic distance. However, there was a significant correlation between geographic distance and island-mainland habitat type, suggesting that the two matrices are collinear and their effects cannot be differentiated. The spatial autocorrelation indicated negative autocorrelation from approximately 17–45 km. These results appear to contrast with those of Muñoz-Fuentes et al. [13] who reported that geographic distance was unlikely to explain the spatial structure of wolf mtDNA haplotypes in a broader study of coastal and central BC. Wolves are highly capable dispersers able to travel > 70 km/day (Mech and Boitani [45]), and it seems unlikely that geographic distance alone can explain the island-mainland structure suggested by the sPCA. In such a situation, we would expect the spatial autocorrelation results to show consistent (and increasing) negative kinship-values with geographic distance. In contrast, the 45–50 distance class that represents wolves in the northern- and southernmost parts of our study, which are farthest apart in geographic distance, showed positive values (or, more likely, no autocorrelation). Multiple interacting factors, including distance, water, terrain ruggedness etc., may affect genetic structure in our study area. Although it is essential to evaluate the possible influence of physiography on the differences observed between island and mainland wolves, the observed correlation between geographic distance and habitat type combined with the physical complexity of the landscape make it problematic and potentially misleading to use linear distances for estimating wolf movement.

Water barriers between the mainland and islands might restrict dispersal and gene flow. For example, captive wolves released on Coronation Island in Alaska did not swim 900 m to nearby habitat with abundant food (Klein [46]). In our study, we reject this hypothesis because we commonly observe wolves swimming among landmasses and distances among islands (including the multiple landmasses used by some groups) are often larger than the distances between islands and the mainland (Darimont et al., *unpublished data*). Immigrants from outside the study area could also influence the observed east–west gradient in genetic profiles. The differentiation seen in YC profiles, for example, may be explained by gene flow from unsampled wolves on the outer islands father west. Similarly, profiles from the UR group, which showed considerable overlap with island wolves, might result from immigration by one or more island wolves with high reproductive success. Furthermore, the presence of intermediate profiles in the north and south of our study area implies an island-mainland gradient. A strict island-mainland dichotomy may thus be simplistic and should be evaluated on a broader geographic scale. Without genetic data from a larger spatial extent, however, we cannot evaluate these hypotheses.

Family group structure might also have influenced our results, especially for long-lived animals for which the genetic influence of one successful breeder can be detected for many generations. Difficulties with amplification of *MS41B* likely reduced our ability to identify male wolves. A possible higher prevalence of females in the sample might nevertheless exacerbate genetic structuring in species where males are more likely to disperse. However, male wolf profiles we assessed showed a similar island-mainland gradient. Observational and tracking data suggest that wolf group size in the study area was ≤ 10 individuals (Darimont [24]), and it appears unlikely that the observed gradient in genetic profiles could be explained by social structure (i.e., wolf pack membership) alone. The identification of 116 individuals in the study area appears reasonable for a sampling period that included 2 litters, winter pup mortality that may exceed 50%, and the likelihood that 20% of individuals would be solitary or extra-territorial dispersers (Mech and Boitani [47]).

Despite the above-mentioned uncertainties, we offer the working hypothesis that the sharp ecological gradient between island and mainland locations, as revealed by the landscape characteristics and the dietary and parasitic data from wolves in our study area, can influence population genetic structure. Although our study must be interpreted with caution, and should be repeated with genetic profiles of higher quality, the results appear consistent with an increasing body of literature reporting genetic differentiation in wolves and other highly mobile species (see Introduction) influenced by ecological and environmental factors. Dispersal rates and gene flow might differ substantially between island and mainland sub-populations, and the extent to which populations are demographically independent could help define management units (Palsbøll et al. [48]) along the Pacific coast.

Associated morphological or other characteristics observed over time might have allowed TEK knowledge holders to recognize these dissimilar wolf forms. Such intra-specific nomenclature is common among indigenous knowledge holders (Turner et al. [49]). Indeed, in adjacent southeast Alaska, the frequency of the black colour phase among wolves killed by trappers is ~50% on the mainland and only ~20% on the islands (Person et al. [50]). Additional morphological differences among wolves of coastal BC might have led to mainland-island classification by local people.

The evolutionary influence of marine resources, which are pronounced on islands in our study area, can be dramatic for terrestrial wildlife. For example, polar bears (*U. maritimus* Phipps, 1774) are thought to have evolved from grizzly bears in peripheral areas where marine resources were abundant (Shields et al. [51]). Moreover, wolves of coastal BC (mainland and island populations) were thought to have diverged from interior populations in part because of marine resource availability in coastal zones (Muñoz-Fuentes et al. [13]). Individuals born in this distinct environment are likely better able to survive and reproduce within, compared to beyond, these conditions.

## Conclusions

Our results indicate the presence of a genetic cline between island and mainland wolves. Although overlap was extensive, the results suggest an east–west gradient in profiles across < 30 km. We hypothesize that adaptive responses to heterogeneity in food resources can influence genetic differentiation. Accordingly, this line of inquiry presents an exciting avenue for future research where marine resources or other components of ecological heterogeneity are present.

## Competing interests

The authors declare that they have no competing interests.

## Authors’ contributions

ELN carried out laboratory work, and contributed to fieldwork, statistical analyses, manuscript writing and revisions. AVS performed statistical analyses and manuscript writing. MSQ supervised the study and helped revise the manuscript. CTD participated in study design, fieldwork, and manuscript writing. PCP contributed to study design, field work and manuscript revisions. HMB created the maps and helped revise the manuscript. All authors read and approved the final manuscript.

## Supplementary Material

Additional file 1 **Map of the study area on the central coast of British Columbia, Canada.** Shown are estimated home ranges of five wolf (*Canis lupus*) social groups. Click here for file

Additional file 2 Multiplex combinations of 14 microsatellite markers for genetic analyses of wolves from the central coast of British Columbia, Canada. Click here for file

Additional file 3 Calculation of amplification and error rates and assessment of null alleles, large allele dropout, and stutter peaks for wolf samples (n = 116) from the central coast of British Columbia, Canada. Click here for file

Additional file 4 **Colour plot of wolf profiles from the central coast of British Columbia, Canada. a)** Individual profiles (n = 116) based on ≥ 10 microsatellite loci. The first axis represents 6.1% of the variation, the second axis 5.1%. **b)** A subsample of individual profiles (n = 18) based on ≥ 5 duplicated loci. Genetic diversity is represented by distance and colour; individuals further apart and/or labelled with more dissimilar colours have more divergent genotypes. The first axis represents 18.8% of the variation, the second axis 13.9%. Click here for file

Additional file 5 **Principal component analysis (PCA) of male wolves from the central coast of British Columbia, Canada, showing island (IS, n = 19) and mainland (MA, n = 9) individuals.** The first axis represents 11.9% of the variation, the second axis 9.7%. PCA is based on the 10 loci retained for final analyses (Table 1 and Additional file 2). Click here for file

Additional file 6 **Eigenvalues from a spatial principal component analysis (sPCA) on 10 microsatellite loci from 116 wolves from the central coast of British Columbia, Canada.** Positive values (left side) represent global structures and negative values (right side) show local patterns. Tests for local and global structure revealed the presence of one global structure, which was subsequently interpreted. Click here for file

Additional file 7 **Spatial autocorrelation analysis of wolf samples (n = 116) from the central coast of British Columbia, Canada, using 5 km distance classes.** The Y axis shows the kinship coefficient (r), and U and L are the upper and lower limits for the 95% confidence interval of no spatial structure occurring in the data set after permutation (n = 999). Error bars show the 95% confidence interval around r as determined by bootstrap resampling (n = 999). Click here for file
