# Supplementary material for: Population genetic structure of gray wolves (Canis lupus) in a marine archipelago suggests island-mainland differentiation consistent with dietary niche
Source: BMC Ecol. 2014 Jun 10;14:11. doi: 10.1186/1472-6785-14-11 (PMC4050401; doi:10.1186/1472-6785-14-11)
Supplement: Additional file 1 — Map of the study area on the central coast of British Columbia, Canada. Shown are estimated home ranges of five wolf (Canis lupus) social groups. [file 1472-6785-14-11-S1.doc]

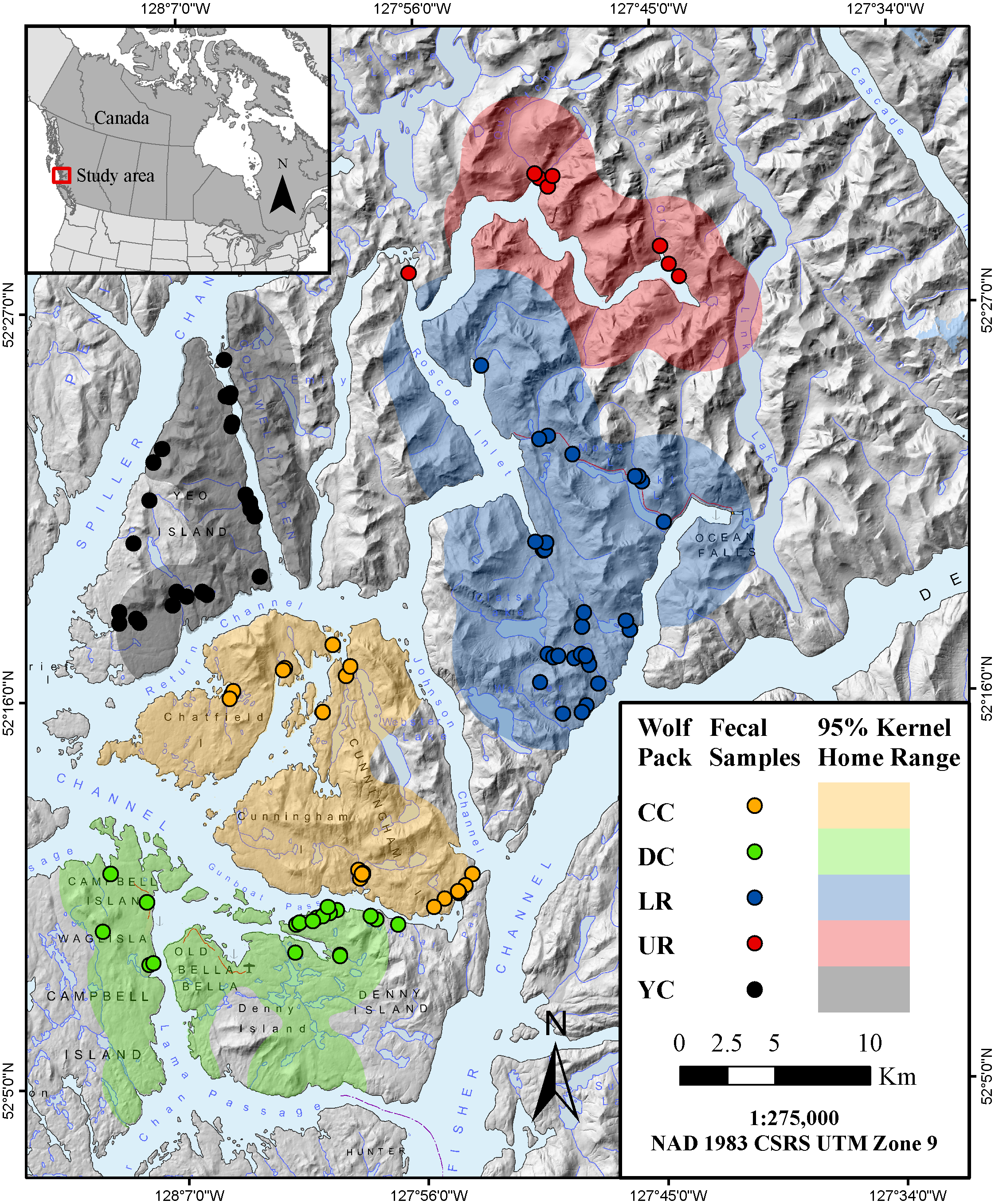


Additional file 1. Map of the study area on the central coast of British Columbia, Canada. Shown are estimated home ranges of five wolf (*Canis lupus*) social groups.
