# Supplementary material for: Population genetic structure of gray wolves (Canis lupus) in a marine archipelago suggests island-mainland differentiation consistent with dietary niche
Source: BMC Ecol. 2014 Jun 10;14:11. doi: 10.1186/1472-6785-14-11 (PMC4050401; doi:10.1186/1472-6785-14-11)
Supplement: Additional file 2 — Multiplex combinations of 14 microsatellite markers for genetic analyses of wolves from the central coast of British Columbia, Canada. [file 1472-6785-14-11-S2.doc]

Additional file 2. Multiplex combinations of 14 microsatellite markers for genetic analyses of wolves from the central coast of British Columbia, Canada.

| **Multiplex** | **Combinations of microsatellite loci** |
| --- | --- |
| A | FH3313, FH2422, FH2001, FH2096 |
| B | FH2017, MS41b, FH2054 |
| C | FH2010, PEZ06 |
| D | PEZ08, FH3725 |
| E | PEZ15, FH2088 |
| F | PEZ19 |
