# Supplementary material for: Population genetic structure of gray wolves (Canis lupus) in a marine archipelago suggests island-mainland differentiation consistent with dietary niche
Source: BMC Ecol. 2014 Jun 10;14:11. doi: 10.1186/1472-6785-14-11 (PMC4050401; doi:10.1186/1472-6785-14-11)
Supplement: Additional file 3 — Calculation of amplification and error rates and assessment of null alleles, large allele dropout, and stutter peaks for wolf samples (n = 116) from the central coast of British Columbia, Canada. [file 1472-6785-14-11-S3.doc]

Additional file 3. Calculation of amplification and error rates and assessment of null alleles, large allele dropout, and stutter peaks for wolf samples (n = 116) from the central coast of British Columbia, Canada.

| **Locus** | **Amplification Rate**†  **(%)** | **Error Rate**§  **(%)** | **Null Alleles** | **Large Allele Dropout** | **Stutter Peaks** |
| --- | --- | --- | --- | --- | --- |
| FH2054 | 93 | 29 | Yes | No | Yes |
| FH2001 | 99 | 33 | Yes | No | No |
| FH2096 | 99 | 23 | No | No | Yes |
| FH2010 | 88 | 26 | Yes | No | No |
| FH2017* | 30 | 21 | Yes | No | Yes |
| PEZ08* | 52 | 19 | Yes | No | Yes |
| MS41B* | 22 | 6 | ** | No | No |
| FH2088 | 99 | 20 | No | No | No |
| FH2422 | 91 | 33 | Yes | No | Yes |
| FH3313* | 50 | 26 | Yes | No | Yes |
| PEZ06 | 95 | 32 | Yes | No | No |
| PEZ19 | 71 | 17 | Yes | No | Yes |
| PEZ 15 | 87 | 16 | Yes | No | No |
| FH3725 | 96 | 29 | Yes | No | No |
| MEAN | 77 | 24 |  |  |  |

†Calculated on all samples that amplified ≥ 9 of 14 loci.

§Calculated on a subset of 50 re-tested samples.

*Excluded from further analyses.

** Locus on haploid Y-chromosome.
