# Supplementary material for: Population genetic structure of gray wolves (Canis lupus) in a marine archipelago suggests island-mainland differentiation consistent with dietary niche
Source: BMC Ecol. 2014 Jun 10;14:11. doi: 10.1186/1472-6785-14-11 (PMC4050401; doi:10.1186/1472-6785-14-11)
Supplement: Additional file 4 — Colour plot of wolf profiles from the central coast of British Columbia, Canada. a) Individual profiles (n = 116) based on ≥ 10 microsatellite loci. The first axis represents 6.1% of the variation, the second axis 5.1%. b) A subsample of individual profiles (n = 18) based on ≥ 5 duplicated loci. Genetic diversity is represented by distance and colour; individuals further apart and/or labelled with more dissimilar colours have more divergent genotypes. The first axis represents 18.8% of the variation, the second axis 13.9%. [file 1472-6785-14-11-S4.doc]

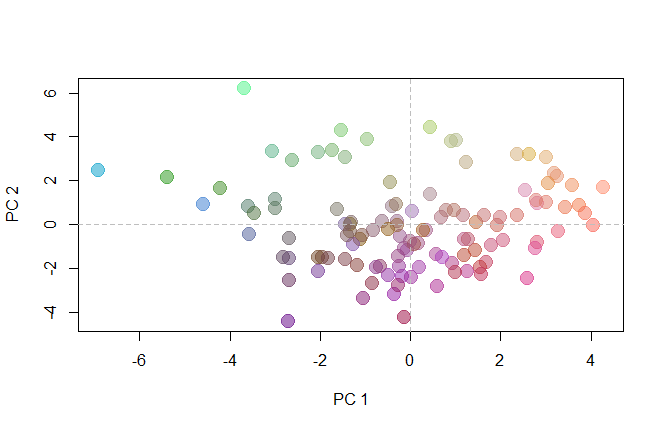


a)


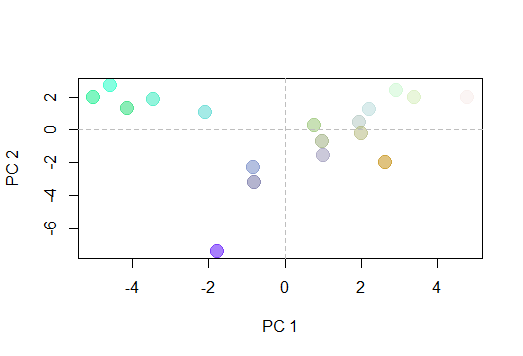


b)

Additional file 4. Colour plot of wolf profiles from the central coast of British Columbia, Canada. a) Individual profiles (n = 116) based on > 10 microsatellite loci. The first axis represents 6.1 % of the variation, the second axis 5.1 %. b) A subsample of individual profiles (n = 18) based on > 5 duplicated loci. Genetic diversity is represented by distance and colour; individuals further apart and/or labelled with more dissimilar colours have more divergent genotypes. The first axis represents 18.8 % of the variation, the second axis 13.9 %.
