# Supplementary material for: Population genetic structure of gray wolves (Canis lupus) in a marine archipelago suggests island-mainland differentiation consistent with dietary niche
Source: BMC Ecol. 2014 Jun 10;14:11. doi: 10.1186/1472-6785-14-11 (PMC4050401; doi:10.1186/1472-6785-14-11)
Supplement: Additional file 5 — Principal component analysis (PCA) of male wolves from the central coast of British Columbia, Canada, showing island (IS, n = 19) and mainland (MA, n = 9) individuals. The first axis represents 11.9% of the variation, the second axis 9.7%. PCA is based on the 10 loci retained for final analyses (Table 1 and Additional file 2). [file 1472-6785-14-11-S5.doc]

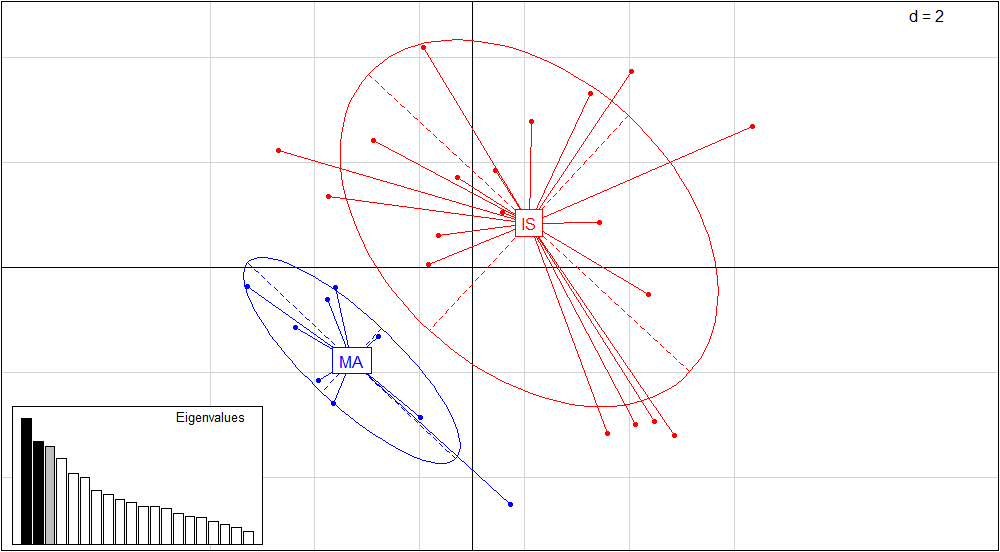


Additional file 5. Principal component analysis (PCA) of male wolves from the central coast of British Columbia, Canada, showing island (IS, n = 19) and mainland (MA, n = 9) individuals. The first axis represents 11.9 % of the variation, the second axis 9.7 %. PCA is based on the 10 loci retained for final analyses (Table 1 and Additional file 2).
