# Supplementary material for: Population genetic structure of gray wolves (Canis lupus) in a marine archipelago suggests island-mainland differentiation consistent with dietary niche
Source: BMC Ecol. 2014 Jun 10;14:11. doi: 10.1186/1472-6785-14-11 (PMC4050401; doi:10.1186/1472-6785-14-11)
Supplement: Additional file 6 — Eigenvalues from a spatial principal component analysis (sPCA) on 10 microsatellite loci from 116 wolves from the central coast of British Columbia, Canada. Positive values (left side) represent global structures and negative values (right side) show local patterns. Tests for local and global structure revealed the presence of one global structure, which was subsequently interpreted. [file 1472-6785-14-11-S6.doc]

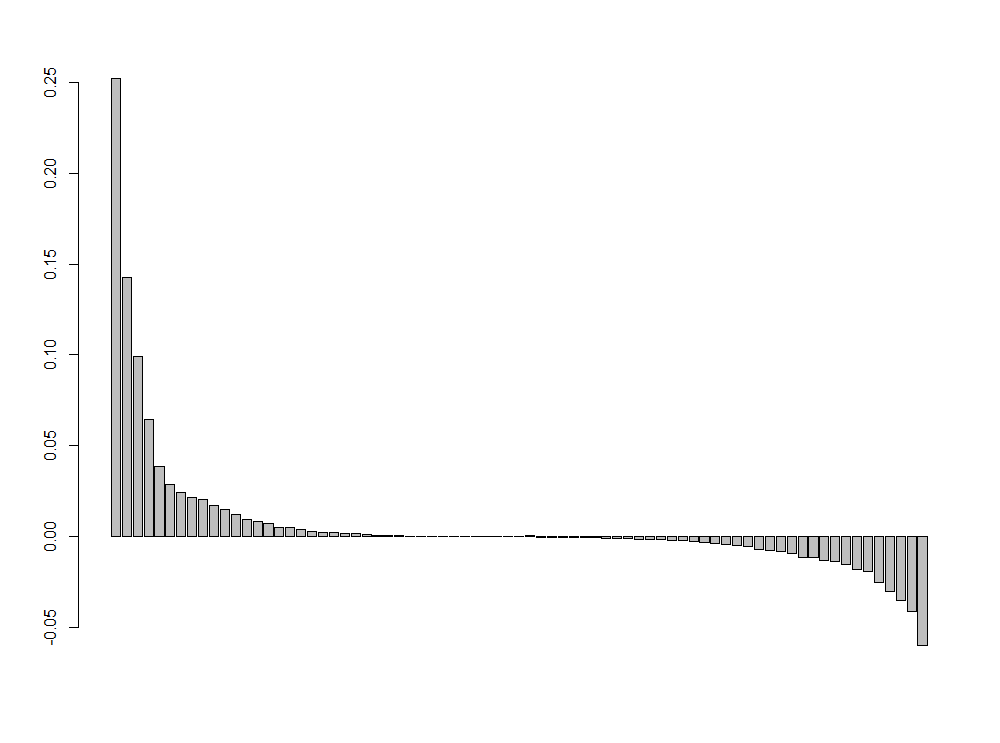


Additional file 6. Eigenvalues from a spatial principal component analysis (sPCA) on 10 microsatellite loci from 116 wolves from the central coast of British Columbia, Canada. Positive values (left side) represent global structures and negative values (right side) show local patterns. Tests for local and global structure revealed the presence of one global structure, which was subsequently interpreted.
