# Supplementary material for: Population genetic structure of gray wolves (Canis lupus) in a marine archipelago suggests island-mainland differentiation consistent with dietary niche
Source: BMC Ecol. 2014 Jun 10;14:11. doi: 10.1186/1472-6785-14-11 (PMC4050401; doi:10.1186/1472-6785-14-11)
Supplement: Additional file 7 — Spatial autocorrelation analysis of wolf samples (n = 116) from the central coast of British Columbia, Canada, using 5 km distance classes. The Y axis shows the kinship coefficient (r), and U and L are the upper and lower limits for the 95% confidence interval of no spatial structure occurring in the data set after permutation (n = 999). Error bars show the 95% confidence interval around r as determined by bootstrap resampling (n = 999). [file 1472-6785-14-11-S7.doc]

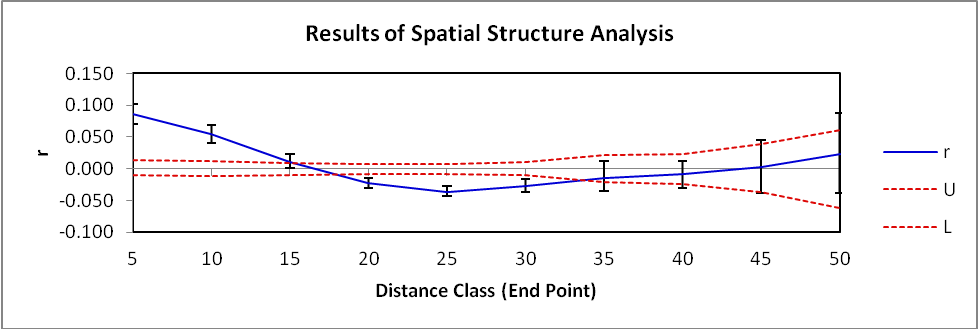


Additional file 7. Spatial autocorrelation analysis of wolf samples (n = 116) from the central coast of British Columbia, Canada, using 5 km distance classes. The Y axis shows the kinship coefficient (r), and U and L are the upper and lower limits for the 95% confidence interval of no spatial structure occurring in the data set after permutation (n = 999). Error bars show the 95% confidence interval around r as determined by bootstrap resampling (n = 999).
